# Agreement between parent and child report of physical activity, sedentary and dietary behaviours in 9-12-year-old children and associations with children’s weight status

**DOI:** 10.1186/s40359-018-0227-2

**Published:** 2018-04-10

**Authors:** Maaike Koning, Astrid de Jong, Elske de Jong, Tommy L. S. Visscher, Jacob C. Seidell, Carry M. Renders

**Affiliations:** 1 Research Centre Healthy Cities, Knowledge Centre for Health and Social work, Windesheim University of Applied Sciences, PO box 10090, 8000 GB Zwolle, the Netherlands; 2 Department of Health Sciences, Vrije Universiteit, Amsterdam, the Netherlands; 3 Pedagogical Studies, Department for Health and Social Work, Windesheim University of Applied Sciences, Zwolle, The Netherlands

**Keywords:** Agreement, Child reports, Parent proxy reports, Health behaviours, Meal patterns, Physical activity

## Abstract

**Background:**

To date, population based surveys aimed at gaining insight in health related behaviour of children have often used either child self-reports or parent proxy reports. It remains unclear however, if surveys using different sources of information from either parents or children are comparable. In addition, (over)weight status of children can lead to under- and over reporting by parents and children as a result of social desirability bias. We aimed at gaining insight in the level of agreement between parents and child reports regarding aspects of certain dietary, physical activity and sedentary behaviours, and whether there are differences in agreement between parents and child reports in healthy-weight and overweight children.

**Methods:**

Weighted kappa was used to determine the level of agreement between child and parent reports on health-related behaviour in 1998 parent-child dyads. We also stratified for weight status of the children. Information on children’s health related behaviours was obtained by parental and children’s questionnaires, and children’s height and weight were measured. Associations between children’s weight status and children reporting less, reporting more and reporting the same amount of health behaviour as their parents were investigated with multinomial logistic regression analysis.

**Results:**

The Cohen’s kappa coefficients ranged from almost perfect agreement for the variable means of transportation, fair for the variables breakfast consumption and frequency of outside play to slight for the variables duration of outside play, frequency and duration of TV/DVD viewing and family dinner. Overweight children were significantly more likely to report less breakfast consumption (OR = 2.6 (95% CI: 1.3 – 5.1)) and lower frequency of outside play than their parents (OR = 1.8 (95% CI: 1.1 – 2.9)).

**Conclusion:**

There can be considerable disagreement between the health related behaviours of children as reported by parents or the children themselves. Based on the present study, it cannot be concluded whether parents’ or children’s reports are more accurate. For future studies, social desirability and recall bias would be best demonstrated in a validation study comparing child and parent self-reports with more objective measures of physical activity and food intake.

**Electronic supplementary material:**

The online version of this article (10.1186/s40359-018-0227-2) contains supplementary material, which is available to authorized users.

## Background

In the context of prevention and management of non-communicable diseases much attention has been paid to the investigation and monitoring of health related behaviour, such as physical activity behaviours, dietary behaviour, sedentary behaviours and sleep behaviours, in both children and adults. These health related behaviours have been known to have an impact on the development of overweight [1–4]. Assessment of these health related behaviours by means of self-administered questionnaires may be influenced by reporting biases. In the case of self-reports of children’s health behaviours, biases from child self-reports and parent proxy reports may be different [5–7]. In order to avoid or minimize such biases there is an increased need for objective measures of food intake (e.g. by use of biomarkers) and physical activity (e.g. by use of movement sensors). However, because of the high costs of such methods, questionnaires are still the most widely used instruments for determining frequency and duration of physical activity and frequency and quantity of food intake, as questionnaires are relatively cheap and efficient instruments for collecting data on a large scale in a relatively short time span.

Both child self-reports and parent proxy reports of children’s health behaviours are used in population surveys, and both types of reporting have limitations for measuring behaviour. To be able to accurately fill out a questionnaire a child must have cognitively reached the level of abstract thinking and be able to conceptualize frequency [5, 8]. Child reports become more reliable with age, from 8 years old children are better at estimating dietary behaviour and physical activity [5, 9–11].

Because of difficulties with children’s cognitive abilities, parent proxy reports instead of child self-reports are often used to determine young children’s dietary behaviour and the amount of physical activity and sedentary behaviour [10–13]. There are also drawbacks with parent proxy reports however, as parents may be more prone to social desirability bias than children [14]. Parents may also not be fully aware of their children’s activities and dietary behaviours, especially if the behaviours take place outside the home. Parent proxy reports are believed to be reasonably accurate if the reported behaviour of the child takes place inside the home [15, 16].

Considering the problems involved in assessing children’s dietary behaviour and physical activity, it is important to study how children and parents report children’s behaviours. Previous studies have shown low agreement between child self-reports and parent proxy reports when measuring food intake. In addition, the few studies assessing agreement between parent and child reports on physical activity show low agreement [6, 7, 9, 17, 18].

It has been known that weight status of children can lead to under- and over reporting of children’s dietary and physical activity behaviour by parents as a result of social desirability bias, socio-cultural biases if overweight is seen as a desirable trait or, if the prevalence is high and overweight is considered “normal” relative to other children [19–24].

It is important to have insight in differences in reports of dietary and physical activity behaviours by parents and by children, for the comparison of results of different studies concerning these health related behaviours, and the translation of these results in implications for policy and practice, but also for recommendations for future data-collection of reports of children’s dietary and physical activity behaviours. Whether differences in these reports are influenced by child weight status is important to know, because accurate assessment of these health related behaviours is essential to finding indications for the development and implementation of interventions of overweight in children.

This study aims to explore 1) the level of agreement between parents and child reports regarding certain health related behaviours, and 2) whether there are differences in agreement between parents and child reports in healthy-weight children and overweight children. We will address these issues using data from the ChecKid study, investigating parent reports and child reports in children of 9-12 years of age.

## Methods

### Study design

The present study used data obtained in the ChecKid study 2012. ChecKid is a repeated cross-sectional study of primary school children aged 4 to 12 years from the city of Zwolle in the Netherlands. ChecKid measures were collected in 2006, 2009, and 2012. The objectives of ChecKid are to investigate trends in overweight and to examine life style behaviours related to childhood overweight and obesity and determinants of these behaviours within families, schools and neighborhoods. ChecKid is part of an integrated approach in which quantitative and qualitative monitoring research and environmental scans support the development, implementation and evaluation of tailored community wide interventions.

From all parents who participated in the study, and from those children who were 12 years and older at the time of data collection, passive consent was obtained. In the Netherlands, for children aged 12 – 16 years, consent from parents as well as consent from children themselves is required for participation in research studies. For children younger than 12 years old parental consent only is sufficient for participation, and for children older than 16 years old no parental consent is needed for participation. Medical ethical approval was obtained from the Medical Ethics Committee of the VU University Medical Centre.

### Study population

A total of 43 primary schools in the city of Zwolle were invited to participate, of which 35 (81%) schools participated. When schools did not want to participate, it was mostly because of other priorities. Participating schools were equally spread over all neighborhoods in Zwolle. When a school agreed to be included in the study, all children attending the school (4-12 years) and their parents were invited to participate by means of letters distributed via the schools. For this study passive consent, which involved distributing a letter to the children’s parents and to children that were 12 years or older describing the study and instructing them to respond only if they did not want (their child) to participate, was required from all parents and from those children that were 12 years old and older. In these letters we included information on the consent procedure, and underlined the possibility for children (and parents) to end participation in the study at any time, even when consent has been obtained. There were 135 (2.2%) parents that did not give consent for participation and 34 (0.6%) children that refused to participate in the anthropometrical measurements. Children without anthropometrical data were excluded from this study because weight status of the child is a crucial variable in this study. Further exclusion criteria for participants were not being proficient in the Dutch language, being older than 12 years of age, and not living in the city of Zwolle.

A total of 3328 children aged 9 to 12 years old from the 6th, 7th and 8th grade completed questionnaires, but 1330 of those children did not have a matched parent report of health behaviours, leaving a sample of 1998 matched parent and child reports. Additionally, anthropometrical measurements of the children (height, weight and waist circumference) were performed. Eligible children included those who had an anthropometric measurement (height and weight), whose parents filled in a self-report questionnaire about the reported behaviours, and who completed a questionnaire themselves. A total of 1998 children aged 9 to 12 years old met these criteria and were included in this study.

### Measurements

#### Anthropometric measurements

Anthropometric measurements were performed during 3 weeks in October and November 2012. Trained students measured body height, weight and waist circumference using a standardized protocol [25, 26]. Height was measured to the nearest 0.1 cm with a stadiometer, and weight was measured to the nearest 0.1 kg with a Seca digital scale. During the measurements, the children wore gym clothing and no shoes. Body Mass Index (BMI) was calculated as weight in kilograms divided by height in meters squared. The children’s age- and sex-specific BMI cut-off points suggested by Cole et al. were used to define thinness, healthy weight, overweight and obesity [27, 28]. We used the term thinness which WHO uses to mean low BMI in adults and adolescents [29]. The international BMI cut offs for child overweight and obesity are based on the adult cut offs of 25 and 30 at 18 years and cover the age range 2-18 years [28]. It would be logical to produce BMI cut offs for underweight or thinness using the same principle. However, presently, no expert guidelines for thinness exist, and the current cut-offs classifying thinness are merely based on supposition [27, 30, 31]. In addition, underweight or thinness does not have the same meaning in adults and children. In adults, underweight or thinness indicates low BMI, and can have serious health consequences and comorbidities, whereas in children underweight is low weight for age and wasting is low weight for height [29]. Cole et al. suggest extending the adult term of thinness to children, meaning low BMI for age [27]. For these reasons, and because the prevalence rates of thinness (9%) and obesity (1.4%) in our study were relatively low, we grouped children who were not overweight and defined them as ‘healthy-weight children’ and grouped children who were overweight and obese and defined them as ‘overweight’.

#### Questionnaires

The ChecKid children’s questionnaire consisted of questions on health-related lifestyle behaviours (diet, physical activity, sleeping habits, sedentary behaviour) and determinants of these behaviours (e.g. home and school environments) and was designed for children aged 9 -12 years of age attending grade 6, 7 and 8 in Dutch primary schools. The children’s questionnaire concerned children’s behaviour during a regular schoolday as we were especially interested in finding indications for interventions that could possibly be implemented or supported in a school setting. The ChecKid parental questionnaire consisted of questions on the same subjects but also included socio-demographic variables such as the child’s age, gender, postal code, ethnicity (assessed by country of birth of both parents) and socio-economic status (SES) (assessed by educational level of parents). Existing validated questionnaires on health behaviour were used for the design of the questionnaires [32, 33].

Because a limited amount of questions was worded in exactly the same way in both the parent and children’s questionnaires, we could only use these questions for our analyses on the level of agreement between parent and child reports. Questions worded in exactly the same way on the parent and child questionnaires were used for the analyses regarding parent-child agreement. For example, we asked children the following questions ‘On how many days do you eat breakfast before going to school during the schoolweek?’ and ‘On how many days do you eat dinner at the dining table with your parents during the schoolweek?’. The corresponding questions for the parents were ‘On how many days does your child eat breakfast before going to school during the schoolweek’ and ‘On how many days do you and your child eat together at the dining table during the schoolweek?’.Children could respond with: (almost) never; 1 day per week; 2 days per week; 3 days per week; 4 days per week; 5 days per week, and the corresponding response categories for parents were: 0 or < 1; 1; 2; 3; 4; 5 days in a regular school week. For the exact questions used see Additional file 1. 

#### Health behaviours

We investigated the level of agreement between parent and children reports with respect to five important health related behaviours: breakfast consumption; family dinner; outside play; means of transportation to school and TV/DVD viewing. Outside play was used as indicator of the child’s physical activity, and TV/DVD viewing was used as an important indicator of sedentary behaviour. Family dinner and breakfast consumption were used as indicators of the child’s dietary behaviour. As the main purpose of this study was to examine agreement between the reports, we only used five questions which were worded identically in the parental and children’s questionnaires. We were aware that that the examined behaviours were used as indicators of the specific behaviours, and thus may not represent the wider health related behaviour.

Frequency of breakfast consumption on schooldays and frequency of eating a family dinner together at the table on schooldays in both parents and children were used as indicators of meal patterns.

Outside play was used as an indicator for physical activity and was measured by investigating time spent on outside play. Parents and children were asked to report frequency and duration of time (in categories) spent on outside play. Average time per day spent on the behaviour was calculated by multiplying the number of days that the child spent on the behaviour by the mid-category values of duration of the item in 5 categories: < 0.5, 0.5-1, 1-2, 2-3, and > 3 h a day, and dividing this by 5; the number of schooldays per week. The categories ‘2-3 h’ and ‘more than 3 hours’ were combined so that the response categories in the parents’ reports were the same as they were for the children’s reports. Current recommendations for children aged 5 to 17 years are to spend at least 60 min per day on outside play [34]. Therefore, outdoor play was dichotomized as < 60 and ≥ 60 min per day.

TV/DVD viewing was used as an indicator for sedentary behaviour, as TV viewing has been known to be an important determinant for the development of overweight [35]. Parents and children were asked to report frequency and duration of time (in categories) spent watching TV/DVD. Average time per day spent on the behaviour was calculated by multiplying the number of days that the child spent on the behaviour by the mid-category values of duration of the item in 5 categories: < 0.5, 0.5-1, 1-2, 2-3, and > 3 h a day, and dividing this by 5; the number of schooldays per week. The categories ‘2-3 h’ and ‘more than 3 hours’ were combined so that the response categories in the parents’ reports were the same as they were for the children’s reports. Current recommendations for children aged 4 to 17 years are not to use screentime for more than 2 h per day [36, 37]. Thus, TV/DVD viewing was dichotomized as < 2 and ≥ 2 h per day.

Means of transportation to school could be indicated by the following options: cycling; walking; on the back of a scooter; on the back of a bicycle; brought by car; by bus; other.

### Statistical analyses

Statistical analyses were conducted using the PASW 20.0 and Stata 11 (StataCorp, College Station, Texas) software packages. Descriptive statistics were used (mean, standard deviations and percentages) to describe the study sample and the differences in parent reports of the behaviours and child reports of the behaviours.

Level of agreement between parent proxy reports and child-self reports.

To assess the level of agreement between child and parent reports about frequency of breakfast consumption, frequency of family dinner, average duration and frequency of outside play and average duration and frequency of TV/DVD viewing, we compared calculated averages of frequency and duration of the studied behaviours. To do so, the weighted kappa statistic was used. The response categories of these variables are ordinal which means that not every disagreement can be weighted the same; for example, a difference between categories of ‘0 days per week’ and ‘5 days per week’ is a more serious discrepancy than a difference between categories of ‘3 days per week’ and ‘4 days per week’. In this study, we used the non-weighted kappa statistic to determine the level of agreement between child and parent reports on the means of transportation to school, because of the categorical response categories. The non-weighted kappa statistic does not take the extent of disagreement in account, every disagreement is weighted evenly [38, 39]. To classify the strength of agreements the standards of Landis and Koch were used for the kappa coefficients: ≤0 = poor, 0.01–0.20 = slight, 0.21–0.40 = fair, 0.41–0.60 = moderate, 0.61–0.80 = substantial, and 0.81–1.0 = almost perfect [38].

The level of agreement between children and their parents was compared between categories of children’s weight status in stratified analyses. We calculated kappa CI’s for healthy-weight and overweight children and compared these (Table 3).

Level of agreement between parent reports and child reports in healthy-weight and overweight children.

We also explored whether children reported more, less or the same amount of the health related behaviour as their parents. The parent-child dyads were categorized into three categories: 1) children reporting the same frequency or duration of the health related behaviour as their parent; 2) children reporting lower frequency or shorter duration of the health related behaviour than their parent (i.e., less hours or days per week of TV/DVD viewing or outdoor play, or less days on which they ate breakfast and participated in a family dinner); 3) children reporting higher frequency or longer duration of the health related behaviour than their parent (i.e., children reported more hours or days per week of TV/DVD viewing or outdoor play than parents, or more days on which they ate breakfast and participated in a family dinner). Children’s weight status and the reporting categories were explored using multinomial logistic regression analysis. First, crude analyses were performed. Second, adjusted analyses were carried out, controlling for potential confounding effects of gender, SES and ethnicity, weight status and age of the parent.

## Results

### Demographic variables and health behaviours

The study sample consisted of slightly more girls than boys (Table 1). The majority of children were of Dutch origin. Mean age of the children was 10.6 years, ranging from 9 to 12 years. The parental questionnaires were completed most often by the mother (86.0%) and parents’ mean age was 41.7 years (SD 4.7). Of the parents, 11% had a low level of education, 21% a medium level of education and 68% a high level of education. In Table 2 the studied behaviours as reported by children and parents are presented.

**Table 1:** Sociodemographic characteristics of the study population

	Total study sample (*N* = 1998)
Mean age of the child – (SD)	10.6 (0.96)
Gender (% boys)	49.4%
Age of the respondent parent (years); mean (SD)	41.74 (4.70)
Relationship to child of respondent parent	
Mother/female caregiver (%)	86.0
Socio-economic status (%)	
Low	11.3
Middle	20.8
High	67.9
Ethnicity (%)	
Non-Western background	10.1
Weight status child (%)	
Thinness	9.0
Healthy weight	80.0
Overweight	9.6
Obesity	1.4
Weight status respondent parent (%)	
Thinness	1.6
Healthy weight	66.9
Overweight	25.3
Obesity	6.2

**Table 2:** Health behaviours as reported by children themselves and as reported by their parents

	Children (%)	Parents (%)
Health behaviours	N = 1998	N = 1998
Breakfast consumption: daily	95.7%	97.3%
Family dinner		
5 days a school week	77.6%	85.2%
3-4 days a school week	17.3%	13.3%
0-2 days a school week	5.1%	1.7%
Outside play; frequency:		
5 days a school week	48.7%	21.5%
3-4 days a school week	37.2%	48.9%
0-2 days a school week	14.0%	28.6%
Outside play; duration: > 1 h per day	48.2%	56.4%
Television viewing, frequency:		
5 days a school week	55.3%	70.9%
3-4 days a school week	26.1%	16.5%
0-2 days a school week	18.6%	12.6%
Television viewing, duration: > 2 h a day	7.4%	7.9%
Means of transportation to school		
Cycling	76.4%	76.7%
Walking	17.2%	18.1%
Other (by car, bus)	6.5%	5.2%

The percentage of children reporting the same amount of behaviour as their parents was lowest for frequency of outside play (30.1%) and duration of TV/DVD viewing (37.5%), and highest for breakfast consumption (95.1%) and family dinner (71.3%). The percentage of children reporting less than their parents was highest for the duration (44.5%) and frequency of TV/DVD viewing (33.3%) and the duration of outside play (33.3%), and the percentage of children reporting more than their parents was highest for frequency of outside play (53.5%).

We investigated differences by gender, ethnicity and socioeconomic status (SES), and we found a statistically significant difference between girls and boys for the frequency of outside play; compared to boys, girls more often reported a greater frequency of outside play than their parents. A statistically significant effect of SES was found for the frequency of outside play; compared to children of lower SES, children of high SES were more likely to report a higher frequency of outside play than their parents. For the variables frequency of breakfast consumption and frequency of TV/DVD viewing we found a different effect of SES, children of high SES more often reporting the same frequency as their parents. We also found an effect of ethnicity, compared with children of western ethnicity, children of non-western ethnicity were more likely to disagree with their parents on the frequency of breakfast consumption, and report either a higher or lower frequency of breakfast consumption.

### Level of agreement between parent proxy reports and child-self reports

The Cohen’s kappa coefficients ranged from almost perfect agreement for the variable means of transportation (0.82), fair for the variables breakfast consumption (0.33) and frequency of outside play (0.21) to slight for the variables duration of outside play (0.19), frequency (0.19) and duration of TV/DVD viewing (0.16), and family dinner (0.13) (Table 3).

**Table 3:** Kappa for the separate questionnaire items, stratified by weight status of the child

		Child’s weight status		
		Total	Healthy Weight	Overweight
Health behaviour	Number of parent child dyads	Kappa (95% CI)	Kappa (95% CI)	Kappa (95% CI)
Breakfast consumption	1965	0.33 (0.21 – 0.45)	0.27 (0.15 – 0.40)	0.45 (0.22 – 0.66)
Family dinner	1965	0.13 (0.09 – 0.17)	0.11 (0.07 – 0.15)	0.24 (0.10 – 0.39)
Outside play; frequency	1930	0.21 (0.18 – 0.24)	0.21 (0.18 – 0.24)	0.19 (0.10 – 0.29)
Outside play; duration	1917	0.19 (0.16 – 0.22)	0.19 (0.15 – 0.22)	0.22 (0.14 – 0.31)
TV/DVD viewing; frequency	1930	0.19 (0.15 – 0.22)	0.19 (0.15 – 0.23)	0.15 (0.05 – 0.27)
TV/DVD viewing; duration	1930	0.16 (0.13 – 0.19)	0.16 (0.13 – 0.20)	0.10 (0.02 – 0.18)
Means of transportation to school	1930	0.82 (0.80 – 0.85)	0.83 (0.80 – 0.86)	0.76 (0.66 – 0.85)

### Level of agreement between parent reports and child reports in healthy-weight and overweight children

#### Kappa

Level of agreement was also explored by child weight status. As can be seen in Table 3, the level of agreement is not significantly different between children with or without overweight. In four variables (frequency of outside play, means of transportation to school, frequency and duration of TV/DVD viewing) the weighted kappa was higher among healthy-weight children than in overweight children, though this was not statistically significantly different.

#### Logistic regression

Multinominal regression analyses were performed with the reported behaviour categorized in three categories (children reporting the same frequency or duration of the health related behaviour as their parent, children reporting lower frequency or shorter duration of the health related behaviour than their parent, and children reporting higher frequency or longer duration of the health related behaviour than their parent) as the dependent variable and weight status of the child dichotomized as overweight versus healthy-weight as the independent variable. After adjustment for gender, SES, ethnicity, parental weight status, and parents age, overweight children had higher odds for reporting less frequent breakfast consumption than their parents (OR, 2.6; 95% CI 1.3- 5.1), and for reporting lower frequency of outside play than their parents (OR, 1.8; 95% CI 1.1-2.9). Both these results were statistically significant (Table 4).

**Table 4:** Multinomial logistic regressions showing associations between weight status of the child and the child reporting more or less health related behaviour than their parent

		Crude analyses, unadjusted		Adjusted for gender, SES and ethnicity, weight status and age of parent	
		Reporting less than their parent	Reporting more than their parent	Reporting less than their parent	Reporting more than their parent
	N	OR (95%-CI)	OR (95%-CI)	OR (95%-CI)	OR (95%-CI)
Dietary behaviours					
Breakfast consumption	1965	3.2 (1.8–5.7)**	1.1 (0.4– 3.3)	2.6 (1.3- 5.1)*	0.8 (0.3–2.5)
Family dinner	1965	0.9 (0.6–1.4)	0.8 (0.5–1.3)	0.7 (0.5–1.1)	0.6 (0.3–1.1)
Physical activity behaviours					
Frequency of outside play	1930	1.7 (1.1–2.5)*	1.0 (0.7–1.4)	1.8 (1.1–2.9)*	1.2 (0.8–1.8)
Duration of outside play	1917	1.0 (0.7–1.4)	0.9 (0.7–1.3)	1.0 (0.7 – 1.4)	0.9 (0.6 – 1.3)
Sedentary behaviours					
Frequency of TV/DVD viewing	1930	0.9 (0.6–1.2)	0.8 (0.5–1.2)	1.1 (0.7–1.5)	0.9 (0.6–1.5)
Duration of TV/DVD viewing	1930	1.3 (0.9–1.8)	1.2 (0.8–1.8)	1.2 (0.8–1.7)	1.1 (0.7–1.7)

*Abbreviations*: *SES* socioeconomic status, *OR* odds ratios, *CI* confidence interval

Healthy-weight children was set as reference group

*P*-values: * *p* < 0.05 for difference between healthy-weight children and overweight children; ** *p* < 0.001 for difference between healthy-weight children and overweight children

## Discussion

In our study, children more often reported less (frequency or duration of) healthy and unhealthy behaviours than parents did, this especially is true for the variable duration of TV/DVD viewing for which most children (44.5%) reported less than their parents. An exception is frequency of outside play for which most children reported more than their parents (53.5%). In other studies children would report significantly more hours of sedentary activities than their parents, and also greater participation in physical activities [7] and higher intake of sugar-sweetened beverages than their parents [33].

An explanation for the lower report of duration of watching TV/DVD and the higher report of frequencies of outside play of children may be that parents are not always fully aware of their children’s activities during the day and that parents and children may use different definitions/interpretations when filling in a survey. It may also be difficult for children to accurately estimate a behaviour such as TV/DVD viewing, as the daily amount may fluctuate more than is the case for other health behaviours. Though parent proxy reports are believed to be reasonably accurate if the reported behaviour of the child takes place inside the home [15, 16], parental obesity status and/or the extent to which parents perceive information about their child’s diet as a reflection of their child’s weight may compromise reporting accuracy. It has also been suggested that a part of the inaccuracy of children’s or parents’ self-reports is deliberate and might be due to social desirability [10]. In addition, many parents underreport the weight status of their children which may also reflect social desirability and lack of awareness [40].

The present study also shows high percentages of children and parents reporting the same behaviour for both breakfast consumption and family dinner, although the kappa scores are no more than fair. This discrepancy in agreement may be accounted for by children and parents providing matching reports for breakfast consumption and the prevalences of the studied behaviours being high (the prevalence of eating breakfast daily was 97.3% and family dinner was 85.2% as reported by parents). The kappa statistic is generally thought to be a more robust measure than simple percent agreement calculation, since kappa takes into account the possibility of the agreement occurring by chance [41]. Kappa is dependent of the spread of agreement in categories, and sometimes we see discrepancies occur because of this, because while the percentage agreement is the same, the percentage agreement that would occur ‘by chance’ can be higher because of high prevalences of the studied behaviour [42–44]. Therefore, kappa can still be relatively low while, in percentages, most parents and children agree.

To our knowledge, agreement between children and parents reports of dietary factors, such as breakfast consumption and having a family dinner together have not been studied before. Studies that investigated the level of agreement between child self-reports and parent-proxy reports of fruit and vegetable consumption in Dutch children showed slight to fair agreement [6, 17] . In our study, we found slight to fair agreement for child and parent reports of meal consumption. The agreement on the item of family dinner was significantly lower than the agreement on the item of breakfast consumption and it is difficult to find an explanation for this. Perhaps the item of family dinner is more subjective than it seems [45]: in the children’s questionnaire, we ask how often the child eats at the dining table with his/her parents, while in the parents’ questionnaire, the question is aimed at the parent (how often do *you* eat dinner with your child at the dining table?). It is possible that the child mostly eats dinner together with the other (non-respondent) parent. Our results concerning reports of meal consumption comply with other studies finding low agreement between parent and child reports.

Our results concerning physical activity and sedentary behaviour are in line with other research. In a study that measured the level of agreement between parents’ and children’s reports of watching television and engaging in sports/outside activities fair agreement was also found [46]. In addition, another study that determined the level of agreement between parent and child reports of leisure sports and television viewing, found slight agreement on those items [7]. We found slight to fair agreement for the variable outside play and slight agreement for the variable TV/DVD viewing. There may be different reasons for these findings concerning the low agreement for these behaviours. The perception of what outside play exactly entails may differ between children and parents because outside play takes place outside the home. Even though TV/DVD viewing takes place inside the home and may be regulated by parents, many children today have their own television in their bedroom and their screentime behaviour may go unnoticed by parents. For both outside play and TV/DVD viewing, children may have some difficulties remembering and conceptualizing both the frequency and duration of these behaviours.

Because of plausible explanations that children and parents may both be more accurate reporters of children’s’ health behaviours, and that this accuracy may vary for children and parents for different health behaviours, it is possible that in future studies using questionnaires regarding children’s health behaviours, both child and parent reports will be explored [47] since we do not have an objective measurement of the reported behaviours and we therefore cannot say anything about the ‘true’ behaviours. The question remains how to adequately address these different data sources, underlining the need for validation studies..

### Healthy-weight and overweight children

Overweight children were significantly more likely than healthy-weight children to report less frequent breakfast consumption (OR, 2.6; 95% CI 1.3- 5.1), and lower frequency of outside play than their parents (OR, 1.8; 95% CI 1.1-2.9). Other studies found that even among children as young as 9 years old, systematic underreporting of dietary intake and over reporting of physical activity by overweight individuals may occur as a result of social desirability [22, 48–50]. Possibly it is not the children (with overweight) but their parents who were more likely to give social desirable answers [51]. We however did not find that parents consistently reported more favorable scores. Weight status of the child remains a source of influence to consider when measuring certain health related behaviours with questionnaires.

### Strengths and limitations of the study

The study is strengthened by the large amount of parent-child dyads in the total sample, 1998 dyads were eligible for the study which are enough dyads to perform reasonable valid (kappa) analyses [52]. The high participation rates and the equal spread of participating schools across one city in the Netherlands also added to the strengths of this study. Furthermore, the weight status of children was obtained by trained students following a protocol, and the questionnaires of children and parents and anthropometric measurements were completed within the same month, which means that both children and parents reported about the same actual behaviour.

Some limitations of our study can be identified. Even though the participation rates were very high, children from families with low and middle SES were under-represented as was also the case in a previous study using the data from previous ChecKid measurements, implying possible selection bias [53]. Furthermore, we investigated questionnaire items on schooldays only and not on weekend days. It may be informative to compare the levels of agreement between school and weekend days as parents could be more aware of their children’s behaviour during weekends. We grouped overweight and obese children but we might have found different agreements for these subgroups if we had been able to separate the overweight and the obese children, as another study found lower agreement in the obese subgroups [7]. Another possible limitation is the arbitrary cut-offs for the strengths of agreement [52], though the kappa scores found in our study do not differ much from other similar studies [6, 7, 17].

## Conclusion

To date, population based surveys aimed at gaining insight in health related behaviour of children have often used either child self-reports or parent proxy reports to measure these behaviours in children. However, it remains unclear if surveys using different sources of information from either parents or children are comparable. There can be considerable disagreement between the health related behaviours of children reported by parents or the children themselves and weight status of the child may be a factor that can influence this agreement. In addition, questionnaires are susceptible to subjectivity and can be interpreted differently by parents and children. Since we do not have an objective measurement of the reported behaviours and we therefore cannot say anything about the ‘true’ behaviours, it is possible that in future studies regarding children’s health behaviours both child and parent reports will be investigated [44]. The question remains how to adequately address these different data sources, underlining the need for validation studies. For future studies, social desirability and recall bias would be best demonstrated in a validation study comparing child and parent self-reports with more objective measures of physical activity and food intake.

## Additional file

Additional file 1: Questions questionnaire Checkid. Questions from the ChecKid questionnaire. Questions used for this manuscript from the parental and children’s ChecKid questionnaires. (DOCX 20 kb)

## Abbreviations

BMI: Body mass index
CI: Confidence interval
OR: Odds ratios
SES: Socio-economic status
