# Supplementary material for: Agreement between parent and child report of physical activity, sedentary and dietary behaviours in 9-12-year-old children and associations with children’s weight status
Source: BMC Psychol. 2018 Apr 10;6:14. doi: 10.1186/s40359-018-0227-2 (PMC5891979; doi:10.1186/s40359-018-0227-2)
Supplement: Supplementary file 1 — Questions questionnaire Checkid. Questions from the ChecKid questionnaire. Questions used for this manuscript from the parental and children’s ChecKid questionnaires. (DOCX 20 kb) [file 40359_2018_227_MOESM1_ESM.docx]

**Questions used from the parental questionnaire**

15. Can you specify if the following options are applicable to your child?

| On how many days … | Amount of days in a regular schoolweek (Monday through Friday) | | | | | |
| --- | --- | --- | --- | --- | --- | --- |
|  | 0 or < 1 | 1 | 2 | 3 | 4 | 5 |
| …does your child eat breakfast before going to school during the schoolweek? |  |  |  |  |  |  |
| …do you and your child eat together at the dining table during the schoolweek? |  |  |  |  |  |  |

31. The following questions concern activities and leisure time of your child during a regular schoolweek. When answering the questions keep in mind that the questions concern the child’s behaviour in an average week during the past month.

| On how many days in a regular schoolweek does your child: | Amount of days in a regular schoolweek (Monday through Friday) | | | | | |  | How much time does your child spend on these activities? | How many hours per day | | | | | |
| --- | --- | --- | --- | --- | --- | --- | --- | --- | --- | --- | --- | --- | --- | --- |
|  | 0 or < 1 | 1 | 2 | 3 | 4 | 5 |  |  | < 0.5 hour per day | 0.5 -1 hour per day | | 1 - 2 hours per day | 2 - 3 hours per day | >3 hours per day |
| Play outside after school | ****  **🡫** | **** | **** | **** | **** | **** |  |  | **** | | **** | **** | **** |  |
| Watch television or DVD |   🡫 |  |  |  |  |  |  |  |  | |  |  |  |  |

41. Which means of transportation does your child mostly use to get to school?

|  **walking** |
| --- |
|  **riding his or her bicycle** |
|  **on the back of parent’s bicycle/ in a stroller** |
|  **on the back of a scooter** |
|  **brought by car** |
|  **by bus** |
|  **other** |

**Questions used from the children’s questionnaire**

3. **On how many days do you eat breakfast before going to school during the schoolweek (Monday through Friday)?**

|  (almost) never |  3 days per schoolweek |
| --- | --- |
|  1 day per schoolweek |  4 days per schoolweek |
|  2 days per schoolweek |  5 days per schoolweek |

5. **Which means of transportation do you mostly use to get to school?**

|  by bicycle |  on the back of a bicycle |
| --- | --- |
|  walking |  brought by car |
|  on the back of a scooter |  by bus |
|  other, namely: |  |

20.

| **On how many days do you eat dinner at the dining table with your parents during the schoolweek (Monday through Friday)?** |
| --- |
|  never |
|  1 day per schoolweek |
|  2 days per schoolweek |
|  3 days per schoolweek |
|  4 days per schoolweek |
|  5 days per schoolweek |

28.

| **On how many days in a regular schoolweek (Monday through Friday) do you watch television or DVD?** |
| --- |
|  never |
|  1 day per schoolweek |
|  2 days per schoolweek |
|  3 days per schoolweek |
|  4 days per schoolweek |
|  5 days per schoolweek |

29.

| **How much time do you spend on watching television or DVD on a regular school day (Monday through Friday)?** |
| --- |
|  I do not watch television |
|  less than half an hour |
|  half an hour to an hour |
|  one to two hours |
|  more than two hours |

33. **On how many days in a regular schoolweek (Monday through Friday) do you play outside after school?**

|  0 days per schoolweek |  3 days per schoolweek |
| --- | --- |
|  1 day per schoolweek |  4 days per schoolweek |
|  2 days per schoolweek |  5 days per schoolweek |

34. **How much time do you spend playing outside on a regular school day (Monday through Friday)?**

|  I never play outside on schooldays |
| --- |
|  less than half an hour |
|  half an hour to an hour |
|  one to two hours |
|  more than two hours |
